# Advancing Federated Learning through Verifiable Computations and Homomorphic Encryption

**DOI:** 10.3390/e25111550

**Published:** 2023-11-16

**Authors:** Bingxue Zhang, Guangguang Lu, Pengpeng Qiu, Xumin Gui, Yang Shi

**Affiliations:** School of Optical-Electrical and Computer Engineering, University of Shanghai for Science and Technology, Shanghai 200093, China; zhangbingxue@usst.edu.cn (B.Z.); 213330769@st.usst.edu.cn (G.L.); 213330798@st.usst.edu.cn (P.Q.); 212260503@st.usst.edu.cn (X.G.)

**Keywords:** federated learning, zero-knowledge virtual machine, homomorphic encryption, verifiability, model privacy

## Abstract

Federated learning, as one of the three main technical routes for privacy computing, has been widely studied and applied in both academia and industry. However, malicious nodes may tamper with the algorithm execution process or submit false learning results, which directly affects the performance of federated learning. In addition, learning nodes can easily obtain the global model. In practical applications, we would like to obtain the federated learning results only by the demand side. Unfortunately, no discussion on protecting the privacy of the global model is found in the existing research. As emerging cryptographic tools, the zero-knowledge virtual machine (ZKVM) and homomorphic encryption provide new ideas for the design of federated learning frameworks. We have introduced ZKVM for the first time, creating learning nodes as local computing provers. This provides execution integrity proofs for multi-class machine learning algorithms. Meanwhile, we discuss how to generate verifiable proofs for large-scale machine learning tasks under resource constraints. In addition, we implement the fully homomorphic encryption (FHE) scheme in ZKVM. We encrypt the model weights so that the federated learning nodes always collaborate in the ciphertext space. The real results can be obtained only after the demand side decrypts them using the private key. The innovativeness of this paper is demonstrated in the following aspects: 1. We introduce the ZKVM for the first time, which achieves zero-knowledge proofs (ZKP) for machine learning tasks with multiple classes and arbitrary scales. 2. We encrypt the global model, which protects the model privacy during local computation and transmission. 3. We propose and implement a new federated learning framework. We measure the verification costs under different federated learning rounds on the IRIS dataset. Despite the impact of homomorphic encryption on computational accuracy, the framework proposed in this paper achieves a satisfactory 90% model accuracy. Our framework is highly secure and is expected to further improve the overall efficiency as cryptographic tools continue to evolve.

## 1. Introduction

Nowadays, machine learning has become an indispensable tool in many fields, but data privacy and security issues are the main reasons preventing its widespread deployment [1]. Federated learning, as one of the three main technological routes for privacy computing, focuses on protecting data privacy and realizing the availability but invisibility of data [2].

Unlike traditional centralized machine learning processes, federated learning protects the data privacy of learning nodes. It utilizes the ideas of local computing and gradient aggregation to enable multiple participants to jointly construct a model with global performance.

The basic federated learning framework includes a central aggregator and multiple distributed learning nodes. The aggregator controls the entire training process, including managing learning nodes, aggregating gradients, and maintaining the global model. Before each round of training begins, the aggregator broadcasts the current global model to the learning nodes. The learning node utilizes its private data to execute training algorithms locally, generate model update gradients, and then submit them to the central aggregator. After collecting gradient information from multiple learning nodes, the aggregator generates a new global model based on a certain aggregation strategy [3]. This process will undergo multiple iterations until the predetermined number of training rounds or convergence conditions are reached.

However, due to its distributed nature, the federated learning process may be affected by malicious nodes. For example, distributed nodes may forge false data or provide inaccurate model training results to the aggregator [4], which can seriously degrade the accuracy of machine learning models.

Several recent studies have proposed introducing zero-knowledge proof techniques for federated learning frameworks [5]. They encode the distributed machine learning process as a series of arithmetic and Boolean circuits. Distributed nodes generate verifiable zero-knowledge proofs along with local models. This approach allows the aggregator to verify the integrity of the machine learning process before aggregating a new global model.

A common problem with these solutions is that developers need to learn about cryptography and develop zero-knowledge circuits to accommodate different classes of machine learning protocols [6]. This process is extremely challenging for complex machine learning protocols. In the last two years, the emergence of the zero-knowledge virtual machine (ZKVM) has greatly simplified the design of circuits for developers. The ZKVM is a zero-knowledge virtual environment deployed on a computer that has the capability to generate proofs for arbitrary computations and subsequently verify them on the fly [7].

In this paper, we introduce the ZKVM into federated learning. We construct the ZKVM on all the nodes, where the learning nodes are created as the provers in the ZKP process, and the aggregators are the verifiers. First, in order to demonstrate the universality of the proposed framework, we implement several mainstream machine learning algorithms in ZKVM and compare the verification costs. Second, considering that learning nodes usually do not have high-performance hardware resources, we discuss the memory required for verifying large-scale machine learning tasks. We find that the framework proposed in this paper is able to realize the verification of arbitrary large-scale computational tasks on smaller devices. Finally, we analyze the security of the framework under a variety of malicious behaviors and demonstrate that this framework is highly secure.

In addition, during the cycle of federated learning, all distributed nodes can easily access the latest aggregated global model, which cannot effectively protect the privacy of the global model [8]. In a practical business environment, we would like to have the final machine learning results available only to the demand side.

We note that homomorphic encryption can solve the data privacy problem under multi-party co-computation [9]. It can preserve the structure of the original data so that it can still perform algebraic operations in the ciphertext state. We implement the BGV fully homomorphic encryption scheme in ZKVM. First, we encrypt the initial model weights so that the distributed computational nodes perform the machine learning process in the ciphertext space and the resulting local model remains encrypted. The actual results can be obtained by only aggregating the global model and decrypting it using the private key.

The contributions of this paper are summarized as follows:

1. We introduce the ZKVM for the first time in the federated learning, which can generate verifiable proofs for machine learning tasks of multiple classes and arbitrary sizes. Meanwhile, we discuss the security of the framework for different malicious behaviors.

2. We implement the BGV fully homomorphic encryption scheme in ZKVM. We encrypt the model information to ensure that federated learning nodes always collaborate in the ciphertext space. This protects model privacy during local training and transmission processes.

3. We propose and implement a new federated learning framework. We conducted extensive experiments on the IRIS dataset to summarize the computational cost and learning performance.

The rest of the paper is organized as follows: Section 2 summarizes the preparatory knowledge on federated learning, zero-knowledge proofs, and homomorphic encryption. Section 3 describes the implementation of the system. Section 4 sets up experiments to evaluate the framework proposed in this paper. Section 5 explains the limitations. Section 6 summarizes the whole paper.

## 2. Background

In this section, we provide a brief introduction to the concepts and cryptographic primitives related to federated learning.

### 2.1. Federated Learning

In 2016, Google Research pioneered the concept of federated learning [10]. Federated learning is essentially a distributed machine learning method, which aims to realize that all parties work together to train machine learning models without exchanging raw data, and to improve the model effect through a series of aggregation algorithms [11]. Federated learning can connect data silos together and effectively build a data ecosystem. It has become one of the important technologies for mining data value in various fields such as healthcare [12], finance [13], and the Internet of Things [14].

However, as data privacy becomes more and more of a concern, federated learning frameworks alone can no longer meet user needs. For example, malicious learning nodes may tamper with the algorithm execution process or submit false learning results, which can directly affect the performance of federated learning. In addition, learning nodes can easily obtain the global model. In practical applications, we would like to obtain the results of federated learning by only one party. Unfortunately, existing research lacks a discussion of the above issues.

In this paper, we combine the state-of-the-art cryptography tools, zero-knowledge proofs, and homomorphic encryption to address the above issues and construct a more complete federated learning framework.

### 2.2. Zero-Knowledge Proofs and Zero-Knowledge Virtual Machine

Zero-knowledge proofs (ZKP) are used to solve trust problems between two parties in scenarios where no third-party trusted institution is involved. The state-of-the-art idea is non-interactive zero-knowledge proofs [15]. The proof process of this scheme requires only one data transfer, which greatly reduces the communication time.

In federated learning scenarios, ZKP can validate the legitimacy of data circulation and manipulation. For example, Ghodsi Z. et al. proposed to generate proofs for local machine learning processes [16]. They encode machine learning protocols into the circuit program. When a learning node returns the local model to the aggregator, it needs to provide a verifiable proof at the same time. The proof is used to verify the integrity of the local machine learning task execution before aggregating the global model. However, designing machine learning protocols directly based on circuit languages is difficult due to complex cryptographic principles. For example, the federated learning framework proposed by Abla Smahi et al. based on zk-SNARKs circuit language is only applicable to the federated support vector machine [17].

In this paper, we introduce the ZKVM for the first time to address the above problem. The ZKVM is a virtual machine that runs trusted code and generates verifications of the output [18]. It is generalized in that it lowers the development threshold for zero-knowledge circuits and is able to generate proofs for arbitrary applications or computations on the fly.

The existing ZKVMs are mainly classified into three types: mainstream, EVM-equivalent [19], and ZK-optimized, and their differences are shown in the Table 1.

This article is based on the popular RISC Zero project. RISC ZKVM is essentially a verifiable virtual machine that operates similarly to a real embedded RISC-V microprocessor [20]. The RISC ZKVM takes care of the underlying cryptography and supports the provision of proofs to arbitrary applications that can run on the RISC-V architecture. Developers need only focus on building the federated machine learning process.

**Figure 1:**
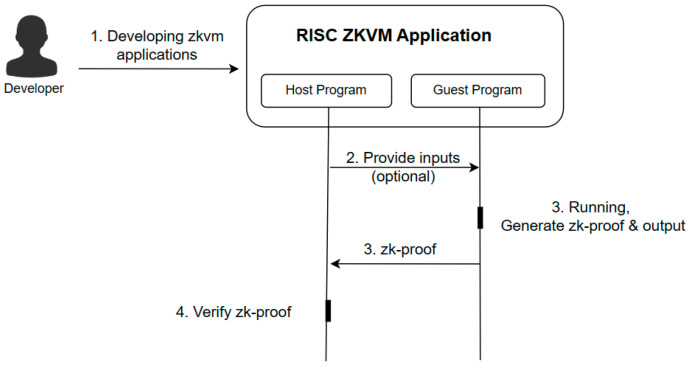
Components of RISC ZKVM application.

As shown in Figure 1, the RISC ZKVM application consists of a host program and a guest program. The host program can provide input to the guest as needed. The guest program generates a zk-proof after execution. Anyone in possession of a copy of the proof can verify the execution of the guest program and read its publicly shared output.

We provide a brief description of how ZKVM guarantees computational integrity. The proof system of RISC Zero is built in terms of an execution trace and several constraints that enforce checks of computational integrity. When performing a machine learning task, the execution trace is a record of the full state of the machine at each clock cycle of the computation. They represent the running state of the processor and effectively check the integrity of RISC-V memory operations. Constraints are low-degree polynomial relations over the values of the constraints. The execution trace is valid if and only if each constraint evaluates to 0. For example, (k)(k − 1) = 0 enforces k to be either 0 or 1. These constraints enforce that the execution of ZKVM is consistent with the RISC-V Instruction Set Architecture (ISA) [21].

In summary, thousands of constraints are first used to enforce the integrity of the RISC-V ISA. Then, at a higher level, constraints are used to enforce that each phase of the machine learning program in ZKVM performs as required.

The ZKVMs are the future, enabling developers to focus on the design of the application itself without paying too much attention to circuits [22]. Constructing ZKVMs on distributed federated learning nodes bridges the gap between zero-knowledge proofs and machine learning programs, making verification of complex machine learning tasks possible.

### 2.3. Private Set Intersection

Private Set Intersection (PSI) is a privacy-preserving protocol designed to compute the intersection of two parties without revealing their private sets. It also can output the result of a function f computed on the intersection [23].

Secret sharing (SS) and oblivious transfer (OT) are the two key techniques used to construct the PSI protocol. SS requires each participant to secretly divide their input data into data shards and distribute them to others. All participants use their received data shards for calculation and interaction. OT is a secure protocol that protects the privacy of communication between both parties. The sender encrypts n messages and sends them to the receiver, but the receiver can only decrypt k of them. Meanwhile, the sender cannot determine which messages the receiver has decrypted.

PSI-based protocol allows for broader data collaboration and analysis. Some applications that have been proposed are secure computation of medical data [24], security event information sharing [25], etc.

### 2.4. Trusted Execution Environment

Trusted execution environment (TEE) uses the method of isolating some of the hardware and software resources to build a secure area on a computing device to ensure the protection of sensitive data and operations [26].

TEE implementations are usually based on hardware technology. Therefore, on different system architectures (x86, ARM, RISC-V) (x86, Arm, RISC-V), different software interfaces, and security boundaries need to be designed. In addition, the expensive hardware cost has become one of the barriers to the widespread deployment of TEE.

As shown in Table 2, we have compared several cryptographic protocols in terms of computational complexity and communication cost.

We discard the TEE scheme because the learning nodes are usually built on different system architectures, which requires more effort to consider the combination of hardware and software technologies.

In order to minimize the number of communications between learning nodes and aggregators, we finally chose the HE. The aggregator encrypts the global model so that only a constant number of communication rounds are required to delegate the machine learning task to each learning node. Throughout the entire federated learning process, the global model and gradient information are always calculated in the ciphertext space, and ultimately only the demand side decrypts to obtain the machine learning results.

### 2.5. Homomorphic Encryption

Homomorphic encryption is a special form of encryption that allows algebraic operations to be performed directly on the ciphertext and the result of the computation remains the ciphertext. It is truly a fundamental solution to the problem of confidentiality when delegating data and its operations to a third party [27].

Depending on the ciphertext operations supported, they can be categorized into semi-homomorphic schemes and fully homomorphic schemes. Semi-homomorphic schemes refer to those that support only additive, multiplicative, or a limited number of full homomorphic operations [28]. This is far from sufficient for complex machine learning tasks. It was not until 2009 that Gentry et al. proposed a strictly full homomorphic encryption scheme (FHE) for the first time [29], which provides a broader application prospect for data privacy protection and secure computing.

Currently, the mainstream fully homomorphic encryption schemes include BGV, BFV, GSW, CCKKS, etc.

BFV is similar to BGV, and both of them need to solve the problem of ciphertext dimensionality expansion brought by homomorphic multiplication through key switching. Meanwhile, the BGV scheme needs to control the noise growth by using modulus switching [30]. Unlike the above schemes, the ciphertext form of GSW is a matrix [31]. It does not have the problem of ciphertext multiplication dimension growth. GSW is theoretically simpler, but the performance is not as good as the BGV and BFV. The subsequent TFHE, FHEW, etc., are based on GSW optimization. In order to perform operations on floating-point numbers, Cheon et al. proposed the CKKS scheme to generate approximate results [32].

Suitable FHE algorithms should be selected according to different scenarios. In machine learning, performance, and accuracy are the main factors to be considered. BGV is considered to be the most efficient scheme among the current algorithms. In addition, BGV maps integers to polynomials, which can satisfy the requirement of computational accuracy.

Therefore, we introduce the BGV scheme for global model privacy protection in the federated learning process. First, the developer encrypts the initial model weights and sends them down to the learning nodes. Then, the learning node performs the machine learning task and submits model updates in the ciphertext state. Even if a malicious node or external attacker steals the machine learning results, it cannot obtain meaningful model information. This approach ensures the privacy of federated learning results during computation and transmission.

We implemented the widely used asymmetric cryptography scheme BGV in ZKVM, and the security of this scheme is based on the ring learning with errors (RLWE) problem [33]. Its plaintext space is defined as $R_{p}=Z_{q}\frac{\left[X\right]}{<\emptyset_{m}\left(X\right)}>,$ where $\emptyset_{m}\left(X\right)$ is an m-dimensional cyclotomic polynomial, *p* is a prime number, and *q* is a large integer. This article sets the basic parameters *m*, *p*, and *q* of BGV as 16, 33554432, and 1073741824, respectively.

The steps of the BGV scheme are briefly described below:

– $BGV.Setup\left(1^{\lambda }\right)$: Input security parameter λ, and output program parameter params.
– $BGV.KeyGen\left(params\right)$: Input the params and output the public key pk as well as the private key sk.
– $BGV.Enc_{pk}\left(msg\right)$: Input the message $msg\in R_{p}$ and output the encrypted message $c\in R_{q}$. This ciphertext consists of two parts, $c=c\left[0]+c[1\right]$.
– $BGV.dec_{sk}\left(c\right)$: Input ciphertext $c\in R_{q}$. Output plaintext message msg.
– $BGV.Add\left(c_{a},c_{b}\right)$: Let $c_{a}$ and $c_{b}$ be the encrypted message of *a* and *b*. Homomorphic addition requires only the addition of the corresponding components:
  (1) $$c_{a}+c_{b}=\left(c_{a}\left[0\right],c_{a}\left[1\right]\right)+\left(c_{b}\left[0\right],c_{b}\left[1\right]\right)=\left(c_{a}\left[0\right]+c_{b}\left[0\right],c_{a}\left[1\right]+c_{b}\left[1\right]\right)=c_{a+b}$$
– $BGV.Mul\left(c_{a},c_{b}\right)$: Homomorphic multiplication is computed via the tensor product of the ciphertext vectors and causes the length of the ciphertext to grow exponentially:
  (2) $$c_{a\cdot b}:=c_{a}\;⮾\;c_{b}:=\left(c_{a}\left[0\right]c_{b}\left[0\right],c_{a}\left[0\right]c_{b}\left[1\right]+c_{a}\left[1\right]c_{b}\left[0\right],c_{a}\left[1\right]c_{b}\left[1\right]\right)$$

In order to solve the efficiency problem caused by the growing dimensionality of the ciphertexts, Zvika Brakerski et al. proposed a method called the “relinearization”. The decryption operation of a long ciphertext $C_{1}$ with a secret key $S_{1}$ is converted into a short ciphertext $C_{2}$ that is decrypted by a different secret key $S_{2}$ [34]. In a recent study, Hiroki Okada et al. proposed that arbitrary binary functions can be realized between ciphertexts by polynomial interpolation [35].

## 3. System Design

This section provides a detailed description of the proposed framework and workflow.

### 3.1. System Overview

In this paper, we construct ZKVM on all federated learning nodes based on the RISC Zero project.

Considering the cost of zk-proof generation and verification, we only implement the machine learning algorithm as a guest program and generate zk-proofs for it. Eventually, when the aggregator receives multiple model updates and proofs, it will use the verified model updates to aggregate a new global model.

**Figure 2:**
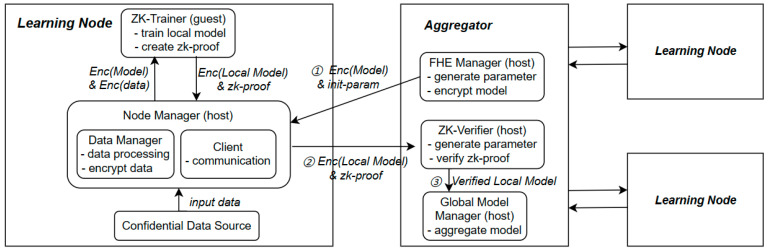
The proposed federated learning framework.

As shown in Figure 2, we describe the framework in terms of two components of federated learning. The local view includes the components running on the distributed learning nodes. The global view considers the central aggregator that communicates with all learning nodes.

**Figure 3:**
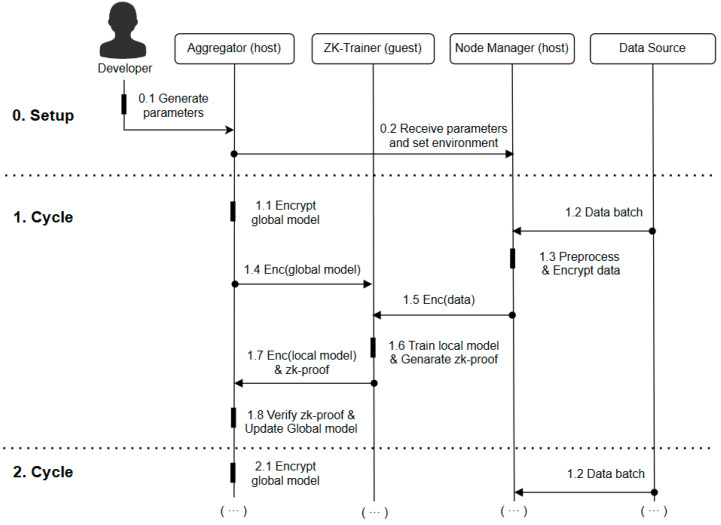
Workflow.

The federated learning process can be summarized in three steps. 1. Learning nodes read encrypted model weights and initialization parameters. 2. Learning nodes perform training tasks to output the ciphertext local model and zk-proof. 3. The aggregator uses the verified local model to aggregate a new global model. The detailed workflow is shown in Figure 3.

(1) Aggregator: The aggregator consists of three components, ZK-Verifier, FHE Manager, and Global Model Manager. It is assumed that the aggregator is a trusted node managed by the task initiator; the aggregation process does not need zero-knowledge proof. In order to reduce the cost of generating proof, all three components are implemented as host programs.

FHE Manager: It is responsible for initializing the BGV scheme. Homomorphic encryption of the global model makes the model information invisible during transmission and local learning.

ZK-Verifier: It is responsible for generating ZKP public parameters such as asymmetric keys, which are used to generate proofs for learning nodes during the machine learning process. It receives and verifies the proof submitted by the learning node.

Global Model Manager: Receiving model updates from all learning nodes, the verified local models are aggregated to produce a new global model.

(2) Learning Nodes: The learning node consists of three components, Data Source, Node Manager, and ZK-Trainer. A learning node becomes a malicious node in a federated learning task when it is attacked. In order to validate the local models submitted by the learning nodes, we implement the zk-Trainer component as a guest program.

Data Source: It is responsible for the storage and management of local privacy data.

Node Manager: It is responsible for the interaction between components. It implements a client that receives ZKP and FHE public parameters from the aggregator and initializes the local execution environment. The data management module reads training data from the data source, preprocesses, and encrypts it. Finally, the local model updates are forwarded to the central aggregator via Client.

ZK-Trainer: The machine learning task is implemented as a zero-knowledge computation, using encrypted global model and data as input. In addition to outputting the local model, zk-trainer generates a proof for the integrity of this local training process.

### 3.2. Workflow

As shown in Figure 3, the workflow of the system consists of a setup phase and a repeating update cycle. For simplicity, the aggregator is represented as a single component.

Setup: In the setup phase, the developer deploys the ZKVM application on all the nodes and initializes the parameters of the BGV scheme. In the federated learning process, the ZK-Trainer is created as the prover and the aggregator as the verifier.

Cycles: At the beginning of each update cycle, the aggregator first homomorphically encrypts the global model (1.1). The node manager of the learning node reads private data from the local data source (1.2). The data are cleaned and sensitive information is removed before homomorphic encryption (1.3). The zk-trainer reads the latest global model from the aggregator (1.4) and receives the processed training data from the node manager (1.5). The zk-trainer performs the zero-knowledge machine learning task to generate the local model and proof (1.6). The proof is sent to the aggregator (1.7) along with the local model. The aggregator first validates the proof with the validation key and then updates the global model with the validated local model (1.8). In successive update cycles, the learning nodes always update the global model in a ciphertext state, thus enabling the protection of global model privacy. At the end of federated learning, only the developer decrypts the global model using the FHE private key to obtain the real machine learning results.

## 4. Experiments

This section comprehensively evaluates the proposed framework through experiments.

First, we analyze the framework in terms of universality, flexibility, and security. Secondly, we designed the federated learning task and measured the required computational cost and model performance. The detailed experimental environment is shown in Table 3.

### 4.1. Framework Analysis

This section analyzes the proposed framework in terms of universality, flexibility, and security.

#### 4.1.1. Universality

As shown in Table 4, we summarize the recent research.

Most of the articles utilize circuit languages such as zk-snarks to generate verifiable proofs for the training process of learning nodes. To further implement the incentives, Heiss J. et al. implemented the verification process as smart contracts on the blockchain using zokrates [37]. However, in these studies, developers need to learn specific zk languages and redesign the machine learning algorithms. This development approach severely hinders the integration of applications with zero-knowledge proof techniques. For the first time, we introduce ZKVM in the federated learning framework to achieve zero-knowledge proof for generalized machine learning algorithms.

In order to demonstrate the universality of the framework proposed in this article, we generated proofs for all the machine learning algorithms mentioned above and verified it.

**Figure 4:**
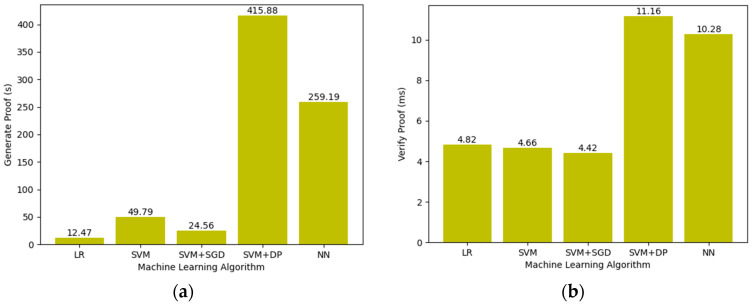
The generation and verification time of proof with different machine learning algorithms: (**a**) generation time of zk-proof; (**b**) verification time of zk-proof.

As shown in Figure 4, we summarize the time cost spent by different machine learning algorithms with the same training samples and computational size. It is obvious that the latter two tasks cost more time to generate and verify the proof. This is since differential privacy (DP) introduces additional stochastic algorithms and noise-adding operations. Similarly, the complex structure and backpropagation of neural networks also lead to an increase in time cost.

#### 4.1.2. Flexibility

The training and inference of artificial intelligence models require significant hardware resources. However, federated learning nodes with private data are usually ordinary users or organizations that lack high-performance devices [40]. We find that there is still a lack of discussion on how to perform zero-knowledge proofs for operations of arbitrary size.

In the framework proposed in this article, the continuation mechanism automatically divides large programs into smaller parts that are independently calculated and proven. These proofs achieve verification of computational integrity for arbitrary programs on memory-limited devices.

To demonstrate the flexibility of the framework, we gradually increase the cycles of the guest programs. The cycle is the smallest unit computed in the ZKVM circuit, analogous to a clock cycle on a physical central processing unit (CPU). The complexity of a program’s execution is measured in cycles as they directly affect the memory, proof size, and time performance of the ZKVM.

**Figure 5:**
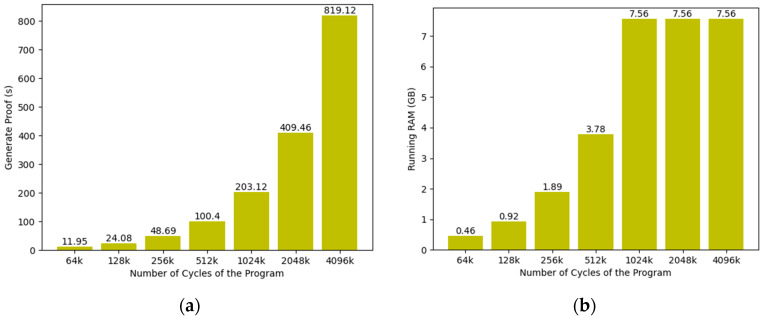
The generation time and running RAM with different numbers of cycles: (**a**) generation time of zk-proof; (**b**) running RAM.

As shown in Figure 5, the time required to generate proofs increases linearly as the cycles of the guest program increase. However, the RAM consumed by the program no longer varies when cycles are greater than 1024k. Only when cycles are less than 1024k, the RAM increases linearly. This is because the framework proposed in this paper splits applications with cycles larger than 1024k into smaller computation parts, which limits the memory consumption to 8G. The split part still needs corresponding CPU time to generate proofs, so it does not affect the generation time of zk-proof.

Overall, the framework is not limited by the size of computing. No matter how long it takes, ZKVM can always generate zk-proof for arbitrary programs under memory constraints.

#### 4.1.3. Security

This section analyzes the security of the framework under different malicious behaviors. The proposed framework in this paper is built on the premise that the training data is trustworthy. This means that the learning node correctly collects and manages local private data.

When the developer designs the federated learning task as a guest program, this program uniquely corresponds to an image ID. The learning node outputs the local model and verifiable proof after executing the guest program. This proof includes information such as image ID, execution trace, and output hash. During this process, malicious nodes may intentionally tamper with the guest program or return incorrect local training results to the aggregator. As shown in Table 5, we analyzed the security of the framework under different malicious behaviors.

Overall, the aggregator’s verification of proof identifies all behaviors of malicious nodes. Aggregating the new global model using only the verified local model guarantees the correctness of the federated learning results.

### 4.2. Federated Learning Performance

**Figure 6:**
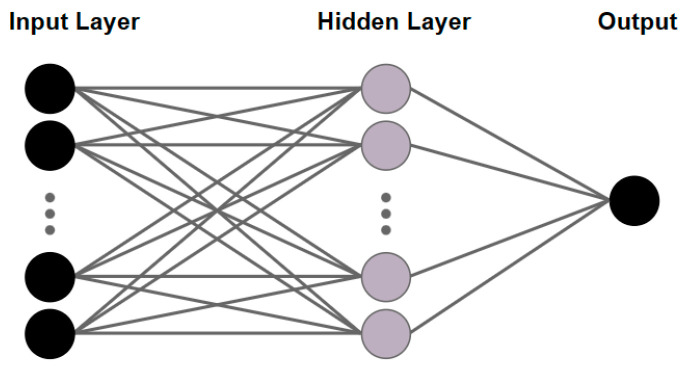
Feedforward neural network with one hidden layer.

The federated learning framework proposed in this paper is generic and applicable to any distributed machine learning task. To gain insight into the performance of this framework, we implemented a feedforward neural network with a single hidden layer [41], whose structure is shown in Figure 6, and the sizes of the input and hidden layers are set to 6 and 10, respectively.

#### 4.2.1. Model Accuracy

We construct a binary classification task after screening the classical IRIS dataset. First, we randomly distribute the dataset on N federated learning nodes, and then perform the local learning process in the ciphertext state, and finally use the average aggregation algorithm to obtain the federated learning results. We measured the model accuracy under different numbers of learning nodes.

**Figure 7:**
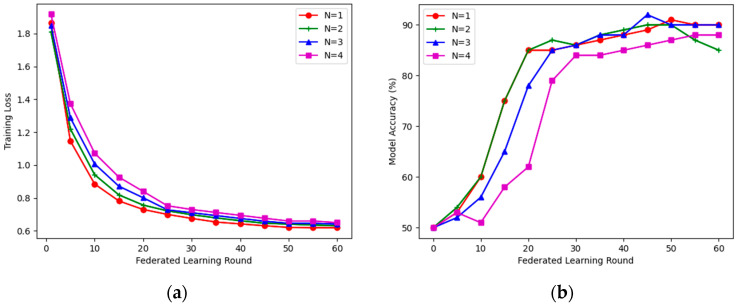
The loss and accuracy of the global model trained with different numbers of learning nodes: (**a**) model loss; (**b**) model accuracy.

Since ZKVM is essentially a virtual machine, only encryption and homomorphic operations have an impact on the model performance. As shown in Figure 7, with different parameter configurations, the global model was able to successfully converge and achieve high accuracy as the number of federated learning rounds increased. For example, the model achieves a 90% accuracy when the number of joint learning nodes is set to either 1 or 3. This is considered a satisfactory result considering the impact of the FHE scheme on computational accuracy and error. The accuracy of the model can be further improved by constructing neural networks with more complex structures or by using more appropriate aggregation algorithms.

#### 4.2.2. Computational Cost

In addition to model performance, computational cost is crucial for the evaluation of federated learning frameworks.

We encrypt each neuron in the model as two polynomials with the highest power of four. Then, forward propagation and backpropagation are performed in plaintext and ciphertext states, respectively, to update the model information. We implemented these machine learning tasks as guest programs in ZKVM that need to be proved. Then, the required computational cost was measured under different federated learning rounds.

**Figure 8:**
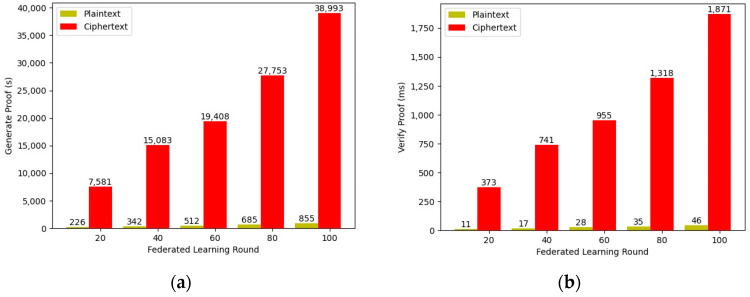
The generation and verification time of proof with different numbers of federated learning round: (**a**) generation time of zk-proof; (**b**) verification time of zk-proof.

We summarize the relationship between the number of federated learning rounds and the generation and verification time of proof in Figure 8. The learning process in the ciphertext state takes more time to generate and verify proof than the plaintext operations. The reason for this is due to a simple plaintext algebraic operation transformed into many operations between polynomials after homomorphic encryption of the model information. Although generating proof takes a considerable amount of time, the verification time is only in milliseconds.

**Figure 9:**
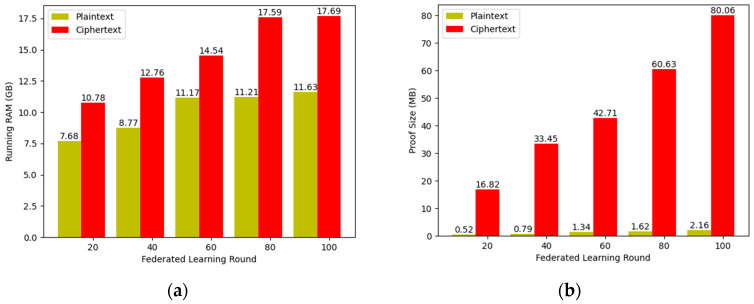
The running RAM and proof size with different numbers of federated learning round: (**a**) running RAM; (**b**) proof size.

As shown in Figure 9, the running memory required by the guest program shows a slower growth as the number of federated learning rounds increases. The reason is that model training is a repetitive arithmetic process, and our guest program based on the rust language implementation has a better memory management mechanism, so the running memory changes less. However, due to the increase in computational complexity, the size of the proof increases linearly with the number of federated learning rounds.

## 5. Limitation

The federated learning framework proposed in this paper verifies the integrity of the machine learning process and protects the privacy of the global model. However, this paper still has some limitations.

First, to protect model privacy, we implement the BGV fully homomorphic encryption scheme in ZKVM. We propose to encrypt the initial model and perform local learning in the ciphertext space. This encryption scheme acting on integers has an impact on the computational accuracy, which reduces the results of federated learning. Second, encrypting model weight as polynomials leads to an increase in computation and requires more time and memory for the validation of machine learning tasks. Nevertheless, as mentioned above, our framework still achieves high model accuracy. With future developments in cryptography, we believe the limitations of this framework can be well addressed.

## 6. Conclusions

In this paper, we propose and implement a federated learning framework based on ZKVM and homomorphic encryption. We implement the federated learning task as a guest program in ZKVM, which verifies the integrity of local model training. In addition, we propose to encrypt the global model, and the learning node outputs the local model in the ciphertext space, which protects the privacy of the global model during training and transmission.

Our framework has broad applicability and can generate zero-knowledge proofs for machine learning tasks of any class and size. At the same time, our framework is highly secure and can effectively identify various behaviors of malicious nodes.

We evaluate the training cost and model performance of federated learning tasks on feedforward neural networks. We find that after implementing machine learning as a ZKP task, it takes more time to generate proof. In contrast, the time required to verify proof is only measured in milliseconds. Furthermore, although homomorphic encryption has an impact on the calculation accuracy, the framework proposed in this paper nevertheless achieves a satisfactory 90% model accuracy.

In the future work, we will work on improving the computational efficiency and accuracy of homomorphic encryption schemes to increase the usability of this framework. In addition, the continuous development of ZKVM technology will help to reduce the time and resources required for generating proofs, which is conducive to further improving the overall efficiency of the framework.

## Figures and Tables

**Table 1:** Classification and differences of ZKVMs.

	Existing Expertise/Tooling	Blockchain Focused	Performant
Mainstream (WASM, RISC-V)	Lots	No	Maybe
EVM-equivalent (EVM bytecode)	Some	Yes	No
ZK-optimized (new instruction set)	No	Yes	Yes

**Table 2:** Computational complexity and communication costs of cryptographic protocols.

Cryptography Tools	Hardware Dependent	Computational Complexity	Communication Rounds	Communication Cost
TEE	Yes	Lower	Lower	Lower
SS	No	Lower	Higher	Moderate
HE	No	Higher	Constant	Lower
OT	No	Higher	Moderate	Lower

**Table 3:** Software and hardware of the experimental environment.

Software and Hardware	Detailed Information
Rust	1.71.1 (ubuntu20.04)
CPU	12 vCPU Intel(R) Xeon(R) Platinum 8255C CPU @ 2.50 GHz
Random Access Memory	16G
Hard Disk	25G
PyTorch	1.11.0
Cuda	11.3

**Table 4:** Zero-knowledge proofs combined with machine learning algorithms. Linear regression (LR); support vector machine (SVM); differential privacy (DP); neural network (NN); stochastic gradient descent (SGD).

Paper	LR	SVM	DP	NN	SGD
[17]		√			
[36]				√	√
[8]	√				
[37]				√	
[38]			√		
[39]	√	√			
Our	√	√	√	√	√

We use √ to indicate which machine learning algorithms have been implemented in the literature.

**Table 5:** Security analysis of frameworks under different malicious behaviors.

Malicious Behavior	Security Analysis
Modify Program	If the program is modified before execution, the image ID in the proof will not match what is expected.
Modify Execution	If the execution is modified, then the execution trace will be invalid. For example, run ZKVM in a debugger and start changing random memory.
Modify Output	If the output is modified, then the output hash will not match the hash recorded in the proof.

## Data Availability

Not applicable.
